# Synthesis and Sintering of Nonstoichiometric (Mo_0.2_Nb_0.2_Ta_0.2_Ti_0.2_W_0.2_)C_x_ Nanoscale Powders for Highly-Dense Ceramics

**DOI:** 10.3390/ma18184293

**Published:** 2025-09-12

**Authors:** Wanxiu Hai, Hai Zhang, Liulin Li, Tong He, Shubo Zhang, Meiling Liu, Yuhong Chen, Youjun Lu, Hailong Wang

**Affiliations:** 1 College of Materials Science & Engineering, North Minzu University, Yinchuan 750021, China; 13575334559@163.com (H.Z.); 17309475821@163.com (L.L.); 15719510669@163.com (T.H.); matinal757791@163.com (S.Z.);; 2 Key Laboratory of Powders & Advanced Ceramics, North Minzu University, Yinchuan 750021, China; 3 Institute of Semiconductor Crystals and Ceramic Materials, Helanshan Laboratory, Yinchuan 750021, China; 4 College of Materials Science & Engineering, Zhengzhou University, Zhengzhou 450001, China

**Keywords:** high-entropy carbide, powder synthesis, nonstoichiometric ratio

## Abstract

Guided by thermodynamic calculations, this study successfully synthesized nonstoichiometric high-entropy carbide (Mo_0.2_Nb_0.2_Ta_0.2_Ti_0.2_W_0.2_)C_x_ (x = 0.875–0.972) nanometer-sized powders using micrometer-sized metal oxides (MoO_3_, Nb_2_O_5_, Ta_2_O_5_, TiO_2_, and WO_3_) and carbon black as raw materials through carbothermic reduction at 1400–1550 °C. The powders synthesized above 1500 °C exhibited a single-phase rock-salt structure with an average grain size as low as 270 nm. TEM analysis confirmed the lattice parameters increased from 0.4378 nm to 0.4395 nm with decreasing carbon content and synthesis temperature. After ball milling, the optimal powder was densified into a (Mo_0.2_Nb_0.2_Ta_0.2_Ti_0.2_W_0.2_)C_0.9_ ceramic block through spark plasma sintering (SPS, 1950 °C/10 min/20 MPa), achieving a relative density of 99.1% and an average grain size of 4.3 μm. This ceramic exhibited remarkable mechanical properties (17.3 GPa Vickers hardness, 25.9 GPa nano-hardness, 524 GPa Young’s modulus, and 4.43 MPa·m^1/2^ fracture toughness) and a relatively low room-temperature thermal conductivity of 8.3 W·m^−1^·K^−1^. This study provides a theoretical basis and technical approach for the preparation of high-hardness and low-thermal-conductivity nonstoichiometric high-entropy carbide ceramics via low-temperature carbothermic reduction and sintering.

## 1. Introduction

Materials in fields such as aerospace, nuclear energy, and high-end equipment manufacturing face increasingly stringent performance requirements. However, the comprehensive performance (i.e., high strength and toughness, high temperature resistance, and good wear resistance) of traditional single-component carbides such as TiC and WC remains unsatisfactory in extreme working environments [1,2]. Against this backdrop, high-entropy carbides (HECs) have emerged as promising multi-component ceramic materials. HECs are characterized by high configurational entropies (which stabilize their multi-component solid solution structures) and significant lattice distortion effects (which provide strength) [3,4]. In recent years, the comprehensive performance of HECs has significantly surpassed that of traditional carbides, especially in extreme service environments [5,6]. Therefore, the development of HECs has rapidly become a research hotspot and frontier.

Nonstoichiometric HECs have recently garnered attention due to their tunable material properties [7]. By precisely controlling the carbon stoichiometry, controllable carbon vacancies can be introduced into HECs [8]. The presence of these carbon vacancies exacerbates lattice distortion and pins dislocations, significantly enhancing the hardness and wear resistance of these materials [9]. Carbon vacancies also introduce local lattice relaxation and micro-zone stress fields, leading to a moderate improvement in fracture toughness [7]. Due to these effects, regulating the concentration of carbon vacancies in HECs can significantly alter their microstructure and performance. For instance, Hossain et al. [10] observed that as the carbon content and chemical state of (HfNbTaTiZr)C_x_ thin films changed, their bonding characteristics and microstructure underwent a significant evolution. Specifically, the material properties gradually transitioned from metallic to ceramic, and ultimately, nanocomposites were formed. Meng et al. [11] studied the mechanical properties of HECs, utilizing first-principles calculations to verify that the presence of carbon vacancies helps enhance their plasticity and toughness. Li et al. [12] prepared (Ti_0.2_V_0.2_Nb_0.2_Mo_0.2_W_0.2_)C_0.875_ nonstoichiometric HECs that exhibited excellent wear resistance, with a low room-temperature friction coefficient of 0.5 and a wear rate of only 10^−6^ mm^3^/N·m. However, the concentration of carbon vacancies needs to be carefully optimized. For example, Tan et al. [13] reported that although the hardness of nonstoichiometric (Zr_0.2_Ti_0.2_Nb_0.2_Ta_0.2_Hf_0.2_)C_0.8_ was comparable to that of fully stoichiometric (Zr_0.2_Ti_0.2_Nb_0.2_Ta_0.2_Hf_0.2_)C, the bending strength of the nonstoichiometric sample significantly decreased by about two-thirds. In summary, controlling the lattice distortion effect precisely by modifying the concentration of carbon vacancies enables the targeted optimization of key HECs properties such as toughness, hardness, and wear resistance. It should be noted that nonstoichiometric HECs exhibit additional comprehensive performance advantages compared to traditional stoichiometric carbides, including excellent oxidation resistance [14], low thermal conductivity, and good dimensional stability [15].

The synthesis of HECs is primarily achieved through the carbothermic reduction of oxides, the direct combination of metal elements with carbon (solid-phase reaction method), or the solid solution method (involving pre-synthesized carbides) [16]. Among these methods, the carbothermic reduction of oxides has been widely adopted due to its significant advantages, including the ability to precisely control the metal–carbon ratio to achieve the target stoichiometric ratio, the low cost and easy availability of raw materials (i.e., metal oxides and carbon), and relatively low reaction temperatures. Consequently, the carbothermic reduction of oxides has become the mainstream synthesis process [17]. However, the HEC powders prepared by this method and the sintered ceramics prepared using these powders are prone to issues such as the uneven distribution of metal elements and the high sensitivity of the carbon vacancy concentration to the synthesis temperature and initial carbon content [18]. Therefore, the influence of synthesis temperature and initial carbon content on the final composition, microstructure, and key properties of nonstoichiometric HEC powders and their ceramics must be investigated in detail. Understanding the relationships between these factors will enable the synergistic control of HEC composition and microstructure, leading to optimized comprehensive performance.

Previous studies on analogous high-entropy carbide (HEC) systems have demonstrated that carbon vacancies within this range (x ≈ 0.85–0.98) can significantly enhance hardness and wear resistance by exacerbating lattice distortion and pinning dislocation motion [19]. However, excessive vacancy concentrations can detrimentally impact fracture strength. The chosen values (x = 0.875, 0.924, 0.972) enable a systematic exploration of this critical trade-off within the Mo-Nb-Ta-Ti-W system, aiming to identify an optimal composition balancing high hardness with adequate toughness. To address the aforementioned issues, this study focuses on the nonstoichiometric high-entropy carbide (Mo_0.2_Nb_0.2_Ta_0.2_Ti_0.2_W_0.2_)C_x_ system. In contrast to prior work on (MoNbTaTiW)C_4.5_, which emphasized sintering and properties [20], this study systematically investigates—guided by thermodynamic calculations—the effects of synthesis temperature (1400–1600 °C) and carbon nonstoichiometry (x = 0.875–0.972) on the formation of single-phase (Mo_0.2_Nb_0.2_Ta_0.2_Ti_0.2_W_0.2_)C_x_ nanopowders. We further examine nanoscale homogeneity and lattice evolution, achieving, via SPS at 1950 °C, a higher relative density (99.1%) and improved mechanical properties, demonstrating the advantage of optimized precursor powder. The mechanical and thermal properties of these ceramic blocks are then evaluated, revealing the unique sintering activity exhibited by the prepared HEC powder. By providing insights into the efficient synthesis of nonstoichiometric HECs, this study offers a theoretical foundation and technical approach for the development of a new generation of ceramic materials resistant to extreme environments.

## 2. Experimental

### 2.1. Preparation of HEC Powders and Ceramic

MoO_3_ (purity > 99.9%, d_50_ of 1.5 μm), Nb_2_O_5_ (purity > 99.9%, d_50_ of 1.0 μm), Ta_2_O_5_ (purity > 99.9%, d_50_ of 2.0 μm), TiO_2_ (purity > 99.9%, d_50_ of 1.0 μm), WO_3_ (purity > 99.9%, d_50_ of 1.5 μm), and carbon black (purity > 99.9%, d_50_ of 4.7 μm) were utilized as raw materials to prepare high-entropy carbide (Mo_0.2_Nb_0.2_Ta_0.2_Ti_0.2_W_0.2_)C_x_ (hereinafter referred to as HEC) powder samples via pressureless sintering according to Equations (1)–(6). All the metal oxide powders used in this study were sourced from Shanghai Yunfu Nanomaterials Technology Co., Ltd. (Shanghai, China).

MoO_3_ + 4 C → MoC + 3 CO (g) (1)

Nb_2_O_5_ + 7 C → 2NbC + 5 CO (g) (2)

Ta_2_O_5_ + 7 C → 2TaC + 5 CO (g) (3)

TiO_2_ + 3 C → TiC + 2 CO (g) (4)

WO_3_ + 4 C → WC + 3 CO (g) (5)

MoC + NbC + TaC + TiC + WC → 5 (Mo_0.2_Nb_0.2_Ta_0.2_Ti_0.2_W_0.2_)C (6)

The detailed chemical compositions of the metal oxides used in this experiment are provided in reference [20]. A series of HEC powders (denoted M1–M10) was prepared using varying reaction temperatures and different initial molar ratios of MoO_3_, Nb_2_O_5_, Ta_2_O_5_, TiO_2_, and WO_3_ to carbon powder, as listed in Table 1. All the (Mo_0.2_Nb_0.2_Ta_0.2_Ti_0.2_W_0.2_)C_x_ powders were prepared using an equimolar ratio of metal atoms, and the carbon content of these powders ranged from x = 0.875 to 1. According to the carbothermic reduction mechanism reported in reference [20], the five metal oxides employed in this work are sufficiently reduced with carbon at 1600 °C to ultimately generate the corresponding monometallic carbides and single-phase high-entropy carbides (achieving complete reduction). The reactions are simplified representations of a multi-step process, wherein CO_2_ may be transiently formed as an intermediate species, although CO is the predominant final gas product under the applied experimental conditions. This predominance arises from the Boudouard reaction becoming thermodynamically favorable above 700 °C, which continuously regenerates CO to maintain a strong reducing atmosphere [21]. It is worth noting that during the preparation of high-entropy carbide ceramics via SPS, appropriately extending the holding time at a relatively low temperature (1600 °C) can effectively promote the densification process, enabling the preparation of high-entropy carbide ceramic blocks with a relatively high density (97.2%). Therefore, in this work, high-entropy carbide powders were prepared through carbothermic reduction within the temperature range of 1400 °C to 1600 °C.

First, the aforementioned five metal oxides and carbon powder were accurately weighed according to the molar ratios shown in Table 1. The weighed raw materials were then placed in a WC-Co cemented carbide ball milling jar, and the corresponding WC-Co cemented carbide grinding balls were added to obtain a ball-to-raw material ratio of 5:1. An appropriate amount of anhydrous ethanol was added as the ball milling medium, and a planetary ball mill was used to ball mill the mixture for 10 h in air. Next, the resulting slurry was dried in an oven at 80 °C. Subsequently, the dried mixture was ground and sieved using a standard 200-mesh sieve. The sieved powder was then placed in a graphite crucible with a diameter of 35 mm and a height of 30 mm, and this small crucible was nested inside a larger (φ130 mm) graphite crucible (xinyulanshan, Shizuishan, China). These nested crucibles were transferred to a vacuum pressureless furnace (ZTY-50-23, Chenrong, Shanghai, China), the furnace atmosphere was set to high vacuum (<10^−2^ Pa), and heating was performed at a constant rate of 10 °C/min to reach the target reaction temperature (1400–1600 °C). The target temperature was maintained for 1 h, and the furnace was then allowed to naturally cool to room temperature. After the sintering reaction, the product was crushed and finely ground in an agate mortar. Finally, the ground powder was sieved through a 200-mesh sieve to obtain the desired high-entropy carbide (Mo_0.2_Nb_0.2_Ta_0.2_Ti_0.2_W_0.2_)C_x_ nanometer-scale powder.

**Figure 1:**
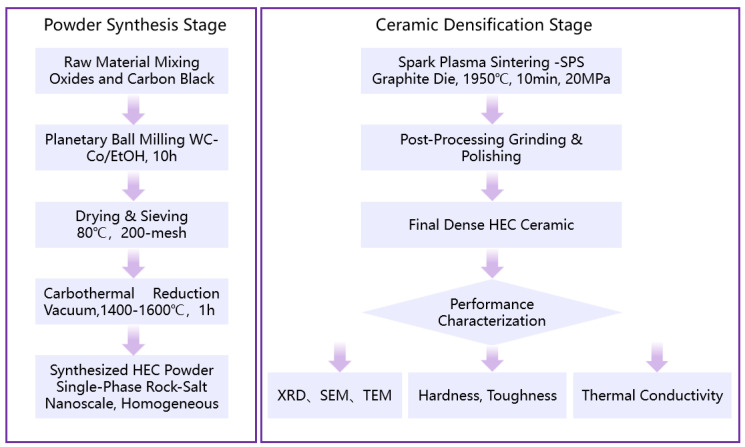
Flowchart for the preparation and performance characterization of HEC ceramic materials.

To prepare a ceramic block, a cylindrical graphite mold with an inner diameter of 25 mm was filled with an appropriate amount of the aforementioned sieved high-entropy carbide powder. Graphite paper was placed between the powder and the inner wall of the mold for isolation. Subsequently, the assembled graphite mold was placed in the center of the SPS system chamber (SPS-4, Chenhua, Shanghai, China). In the initial stage, a minimum pre-pressure of 0.9 kN was applied to fix the mold. Then, a vacuum was applied in the chamber (below 20 Pa). The mold was then rapidly heated to 600 °C at a heating rate of 100 °C/min; simultaneously, the applied pressure was linearly increased from the initial pre-pressure value to 20 MPa. Then, the mold was further heated to 1950 °C using a heating rate of 80 °C/min while maintaining a constant axial pressure of 20 MPa. Finally, the target sintering temperature of 1950 °C was maintained for 10 min under a constant pressure of 20 MPa. After sintering, the heating and pressure systems were turned off, and the sample was allowed to naturally cool to room temperature in the furnace. After cooling, the formed sample was removed from the mold. Next, a surface grinder (4080AHD, YASHIDA, Kunshan, China)was employed to remove the carbon-rich contamination layer formed by contact between the sample surface and graphite, and approximately 1 mm of the surface layer was also ground off to eliminate possible oxidation or heat-affected layers. Subsequently, fine grinding and polishing were performed using diamond grinding pastes with particle sizes of 40 μm, 20 μm, and 5 μm in sequence. Finally, a 0.5 μm diamond polishing suspension was utilized for final mirror polishing to obtain a flat surface to meet the requirements for subsequent microstructural observation and performance testing. The preparation and performance characterization of the HEC ceramic materials are illustrated in Figure 1.

### 2.2. Testing and Analysis

The sintered ceramic blocks were subjected to phase analysis using an X-ray diffractometer (XRD, XRD-6000, Shimadzu, Kyoto, Japan) with a Cu-Kα radiation source (λ = 0.15405 nm), an accelerating voltage of 40 kV, a current of 30 mA, and a scanning rate of 2°/min within the 2θ range of 10° to 80°. The Rietveld refinement analysis of the XRD patterns was conducted using the Jade software (version 6.2, Materials Data Inc.) in conjunction with FullProf Suite 2020.6 software, and the lattice parameters were ultimately determined. Combined with the chemical composition of the ceramic, the theoretical HEC ceramic density was determined. The theoretical density was calculated using the rule of mixtures based on the crystal structure and molecular weight of the constituent phases. The actual density of the sintered ceramic blocks was measured using the Archimedes displacement method, and relative density was calculated based on the theoretical density. The morphology of the powder samples was analyzed using a field emission scanning electron microscope (FESEM, Zeiss supra55, Carl Zeiss, Oberkochen, Germany). Before imaging, the powder samples were dispersed on the conductive tape. For all the sample observations, a secondary electron detector was used, with an acceleration voltage of 5–15 kV and a working distance of 6–9 mm. The SEM micrographs were employed to determine the grain size of the polished ceramic surface using Nano Measurer 1.2 software. To ensure representative statistics, the diameters of at least 200 randomly selected grains were measured. Further microstructural and crystallographic analysis was performed with a high-resolution transmission electron microscope (Talos F200X, FEI, Hillsboro, OR, USA) equipped with a selected area electron diffractometer (SAED) and a high-sensitivity four-quadrant silicon drift detector (Super-X EDS) for elemental analysis. For powder samples, which were ultrasonically dispersed in ethanol and deposited onto a lacey carbon copper grid, observations were carried out at an accelerating voltage of 200 kV. EDS elemental mapping and point analysis were performed under STEM mode with a probe current of approximately 0.7 nA and a probe size of ~0.5 nm to ensure high spatial resolution and sufficient X-ray counts for reliable chemical identification. Energy-dispersive X-ray spectroscopy (EDS, Octane Super, EDAX, Mahwah, NJ, USA) was used for ceramic sample elemental analysis to evaluate the uniformity of the element distribution. A micro-Vickers hardness tester (432SVD, Wobert, Shanghai, China) was employed for hardness testing of the polished surface using an indentation load of 98 N (10 kgf) and a holding time of 10 s. Indentation tests were conducted on at least five different locations on each sample, with the average value taken as the final hardness value. Nanohardness (H) and elastic modulus (E) values were measured using a nano-scratch tester (NST3, Anton Paar, Graz, Austria) for nano-indentation testing. Testing was performed with a maximum load of 4.00 mN, loading/unloading rate of 8.00 mN/min, and a Poisson’s ratio of 0.3.

The fracture toughness of the ceramic blocks was evaluated using the Vickers indentation method, and the fracture toughness of ceramics was calculated according to the Anstis formula (Equation (7)) [22].

(7) $$K_{IC}=0.016{\left(\frac{E}{H}\right)}^{1/2}\frac{P}{c^{3/2}}$$

where *P* is the applied load (N), *E* is the elastic modulus (GPa), *H* is the hardness (GPa), and *c* is the radial crack length.

The thermal conductivity of the ceramic samples was measured using a laser flash apparatus (LFA 467 HT, NETZSCH, Selb, Germany) under a nitrogen protective atmosphere within the temperature range of 25 to 1200 °C.

## 3. Results and Discussion

### 3.1. Thermodynamic Calculations

**Figure 2:**
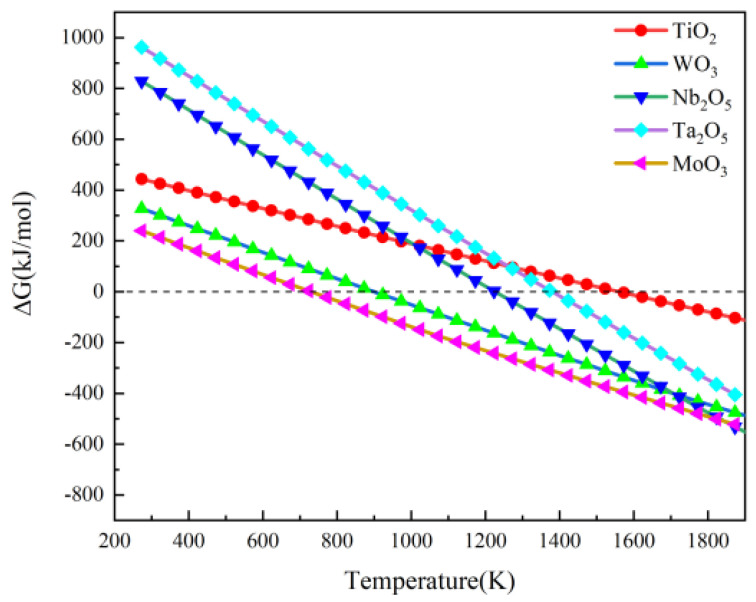
Gibbs free energy change for carbothermal reduction of five oxides with carbon.

The preparation of high-entropy carbide powders from oxide powders involves two key steps. First, each metal oxide needs to be fully reduced to the corresponding carbide through a thorough carbothermic reduction, and the mixed carbide powder should not contain any residual oxygen or free carbon. During the reduction process, oxygen mainly escapes from the reaction system in the form of CO. Second, the generated transition metal carbide powders must exhibit sufficient atomic-level miscibility at high temperatures to form a high-entropy carbide solid solution with a single-phase rock-salt structure [23]. The reduction reactions of the metal oxides employed in this work (MoO_3_, Nb_2_O_5_, Ta_2_O_5_, TiO_2_, and WO_3_) with carbon (C) are shown in Equations (1)–(6). The spontaneity of each reaction at different temperatures was evaluated by calculating the Gibbs free energy ($\Delta_{r}G_{m}^{Ɵ}$) values, providing a theoretical basis for determining suitable reduction process parameters. Figure 2 illustrates the trend of the Gibbs free energy change ($\Delta_{r}G_{m}^{Ɵ}$) with temperature for the carbothermic reduction reactions of the five metal oxides. The initial reduction temperature (the temperature corresponding to $\Delta_{r}G_{m}^{Ɵ}$ = 0 kJ/mol) of MoO_3_ is approximately 500 °C, which is the lowest among the studied oxides [24]. The initial reduction temperatures of the remaining oxides increase in the following order: WO_3_, Nb_2_O_5_, Ta_2_O_5_, and TiO_2_ (which has the highest initial reduction temperature of approximately 1300 °C). As the temperature increases, the $\Delta_{r}G_{m}^{Ɵ}$ values for each reduction reaction decrease, indicating an enhancement in their thermodynamically driven spontaneous progression.

Although this thermodynamic analysis is based on the reactions of single oxides with carbon under standard conditions, it still provides significant guidance for the co-reduction of mixed oxide powders with carbon under low-pressure and high-temperature conditions. According to the calculated $\Delta_{r}G_{m}^{Ɵ}$ values, the five oxides will theoretically be reduced to the corresponding carbides in the order of MoO_3_, WO_3_, Nb_2_O_5_, Ta_2_O_5_, and TiO_2_. When the reduction process is performed under suitable high-temperature conditions, mutual diffusion between the newly formed carbide phases induces the formation of a high-entropy carbide solid solution with a single-phase rock-salt structure, as shown in Equation (6) [25].

In carbothermic reduction reactions involving gaseous products such as CO, reducing the total system pressure is beneficial for lowering the onset temperature (i.e., the critical temperature) of the reaction. This effect stems from a fundamental principle of thermodynamics: driven by Le Chatelier’s principle, lowering the partial pressure of the reaction gas shifts the reaction equilibrium to generate more gas, which reduces the threshold of the Gibbs free energy change required for the reaction to spontaneously proceed [26]. To evaluate the impact of low-pressure conditions, Factsage thermodynamic software (version 7.1) was utilized to calculate the Gibbs free energy change ($\Delta_{r}G_{m}$) with temperature for the five oxide carbothermic reduction reactions under a low pressure of 50 Pa. These values were used to determine the corresponding onset reduction temperatures (the temperature at which $\Delta_{r}G_{m}$ = 0 kJ/mol). The calculation results are summarized in Table 2.

Comparing the initial reduction temperatures obtained under normal pressure (Figure 2) and 50 Pa (Table 2) shows that the low-pressure environment significantly reduces the initial reduction temperature of all oxides [24]. However, these calculation results indicate that the order of the initial reduction temperature (MoO_3_ < WO_3_ < Nb_2_O_5_ < Ta_2_O_5_ < TiO_2_) does not change with pressure. This suggests that the low-pressure conditions mainly affect the magnitude of the thermodynamic driving force of the reaction. However, the relative difficulty of reduction of these oxides (as predicted based on their individual standard $\Delta_{r}G_{m}^{Ɵ}$ values) is not altered.

### 3.2. Characterization of (Mo_0.2_Nb_0.2_Ta_0.2_Ti_0.2_W_0.2_)C_0.9_ Nanopowders

#### 3.2.1. XRD Analysis

**Figure 3:**
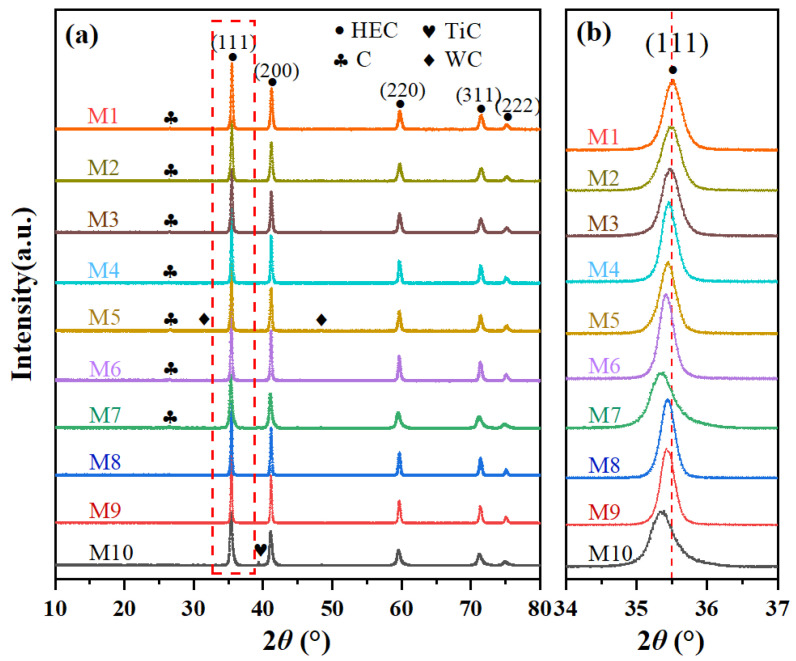
Phase composition analysis. (**a**) XRD patterns of (Mo_0.2_Nb_0.2_Ta_0.2_Ti_0.2_W_0.2_)C_x_ powders and (**b**) enlarged XRD patterns from 34° to 37°.

The XRD patterns of the (Mo_0.2_Nb_0.2_Ta_0.2_Ti_0.2_W_0.2_)C_x_ powder samples M1 to M10 are displayed in Figure 3a. All samples exhibit distinct diffraction peaks at 34.6°, 40.4°, 59.0°, 70.7°, and 74.5°, which are attributed to the (111), (200), (220), (311), and (222) crystal faces of the face-centered cubic (fcc) structure, respectively. This confirms the formation of HEC phases with a rock-salt structure. As observed from Figure 3b, in formulations with the same molar ratio of raw materials (M10–M8, M7–M5, and M4–M2), as the reduction reaction temperature increases, the diffraction peak position of the (111) crystal plane shifts towards higher angles, indicating that the contraction of the unit cell volume corresponds to a decrease in the lattice constant. Notably, no characteristic diffraction peaks corresponding to the initial metal oxides are observed in the XRD patterns, indicating that under the reaction conditions (held at 1400 °C or higher for 1 h), all metal oxides are completely reduced to their respective carbides. This experimental result aligns with Factsage 7.1 thermodynamic calculations, which indicate that under sufficiently high temperatures and ambient pressure, these oxides undergo complete reduction (Figure 2). It should be noted that the XRD patterns of samples M1–M7 show a diffraction peak at 26° corresponding to the (002) crystal face of graphite (PDF#65-6212). This demonstrates the presence of residual carbon, indicating that some of the carbon did not participate in the reduction reaction with these specific formulations. The generation of this residual carbon is primarily attributed to the volatilization and loss of some metal oxides during the reaction process. This leads to a lower number of oxides actually participating in the reduction reaction compared to the design value, resulting in a relative excess of carbon in the system.

The lattice parameter values are presented in the form of ‘a(b)’, where ‘a’ is the refined lattice parameter and ‘b’ in parentheses represents the estimated standard deviation in the last digit. For example, 0.4378(1) denotes 0.4378 ± 0.0001 nm.

In addition, the XRD patterns of M1–M3 and M5 show the characteristic diffraction peaks of tungsten carbide (WC, PDF#72-0097), and those of the M7 and M10 samples show the characteristic diffraction peaks of titanium carbide (TiC, PDF#32-1383). The appearance of the WC phase may be due to the relatively slow diffusion rate of tungsten (W) atoms during solid solution formation preventing the full integration of W into the high-entropy carbide lattice in a timely manner, resulting in local segregation [27]. The presence of the TiC impurity phase is likely related to the fact that the initiation temperature for the carbothermic reduction reaction of TiO_2_ (approximately 1300 °C, Figure 2 and Table 2) is the highest among the five oxides studied, meaning that TiC is formed later than the other carbides. Under the relatively low holding temperature of 1400 °C, the time and driving force may not be sufficient to induce the complete diffusion and dissolution of TiC into the high-entropy carbide phase [28].

In contrast, the XRD patterns of the M8 and M9 samples only exhibit the characteristic diffraction peaks of a single rock-salt HEC structure, with no diffraction signals ascribed to impurity phases or residual graphitic carbon. This indicates that the combination of a higher reaction temperature (1500 °C to 1550 °C) with the addition of 90% theoretical carbon effectively promotes the complete reduction of the five oxides, inhibits the excessive loss of volatile components, and ensures sufficient interdiffusion and solid solution formation between the various carbide components. Consequently, the M8 and M9 samples are HEC powders with pure phase compositions.

The lattice constants of the HEC powder samples (M1–M10) synthesized under different process conditions are summarized in Table 3. Decreasing the reaction temperature and reducing the addition of carbon both lead to an increase in the lattice constant value. At low reaction temperatures, the thermally activated diffusion of metal atoms is more limited, potentially leading to a decline in the uniformity of the metal atom distribution in the HEC lattice and an increase in local composition fluctuations [29]. Consequently, lower reaction temperatures potentially lead to significant lattice distortion. At the same time, as the amount of carbon added to the system decreases, the concentration of carbon vacancies in the formed HEC phase accordingly increases. The presence of these carbon vacancies disrupts the periodic potential field of the lattice, leading to lattice relaxation. An increase in lattice relaxation typically results in lattice distortion, resulting in an increase in the lattice constant [8].

The relationship between lattice parameters and carbon content is crucial in the preparation of high-entropy carbides, but it is not a single pattern: the general trend is that an increase in carbon content leads to an increase in lattice parameters, while reducing carbon addition increases parameters through vacancies and distortions (as stated in the original text). However, evidence shows that negative correlations or non-linear behaviors are more common, which is due to the carbon vacancy contraction effect [30], impurities [31], or system specificity [32]. Therefore, when confirming the stoichiometry of HEC, lattice parameters can be used as a sensitive indicator; however, they must be combined with elemental analysis, vacancy characterization, and condition control (such as reducing oxygen impurities) to distinguish the carbon content effect. Future research should focus on the multi-scale distortion mechanisms of high-entropy systems and establish a unified model to describe this relationship.

#### 3.2.2. SEM Analysis

**Figure 4:**
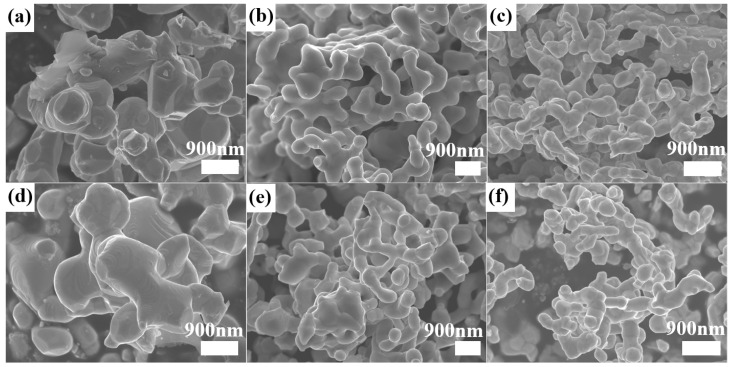
SEM images of (Mo_0.2_Nb_0.2_Ta_0.2_Ti_0.2_W_0.2_)C_x_ samples: (**a**) M5, (**b**) M6, (**c**) M7, (**d**) M8, (**e**) M9, and (**f**) M10.

SEM images showing the morphologies of the M5–M10 HEC powder samples are displayed in Figure 4. The prepared powders consist of agglomerated individual particles at the nanoscale. These particles display a complex coral-like morphology, with clear geometric surface features such as hexagonal platforms and spiral growth steps (typically associated with specific crystal growth mechanisms) [33].

According to statistical analysis of the SEM images, the average grain sizes of the M9 and M8 powders are 0.27 ± 0.14 μm and 0.87 ± 0.4 μm, respectively. Comparing the grain sizes of M7 (M10) (synthesized at a lower temperature) and M5 (M8) (synthesized at a higher temperature) reveals that increasing the reaction temperature significantly promotes the growth of HEC grains, which is consistent with the thermally activated nature of grain growth [34].

#### 3.2.3. TEM Analysis

**Figure 5:**
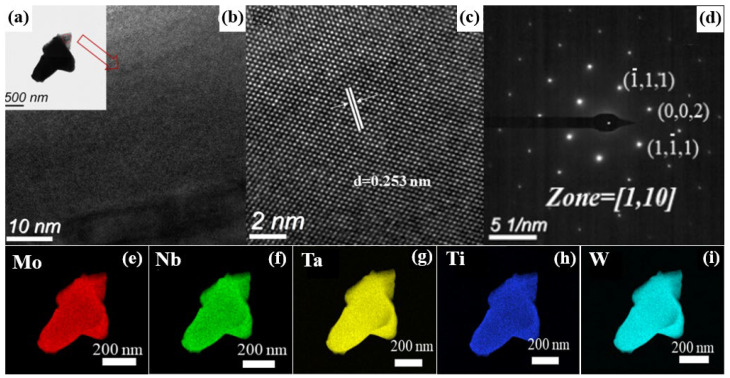
(**a**–**c**) TEM images, (**d**) SAED pattern, and (**e**–**i**) EDS mapping of sample M9.

The crystal structure, nanoscale composition, and homogeneity of sample M9 were characterized using TEM, EDS, and SAED, as shown in Figure 5. The TEM image in Figure 5a shows the morphology of a powder particle, and the high-resolution TEM (HRTEM) image in Figure 5b exhibits clear lattice fringes, confirming the existence of a crystal structure with a well-ordered atomic arrangement. As displayed in Figure 5c, the lattice fringe spacing of this crystal structure is 0.253 nm, from which the lattice parameter is calculated to be 0.4372 nm. This value shows excellent agreement with the lattice parameter of 0.4376 nm determined by XRD analysis for the same sample [35], thereby cross-validating the identified single-phase rock-salt structure and the accuracy of the measured lattice parameter. Figure 5d shows the SAED pattern corresponding to the area shown in Figure 5c. Diffraction spots indexed to the (−1,1,1), (0,0,2), and (1,−1,1) crystal planes of an FCC structure are clearly observed. Notably, no superlattice spots are observed in the SAED pattern, indicating that the five metal atoms (Nb, Ti, Ta, Mo, W) in this sample are arranged in a random, disordered solid solution on the FCC lattice nodes [36]. In addition, the EDS mapping (Figure 5e–i) reveals uniform distributions of the five metal elements (Mo, Nb, Ta, Ti, and W) at the nanoscale, with no obvious elemental segregation or enrichment areas observed. This directly confirms the good compositional uniformity of the prepared HEC powders at the nanoscale.

### 3.3. (Nb_0.2_Ta_0.2_Ti_0.2_W_0.2_Mo_0.2_)C_0.9_ High-Entropy Ceramic

#### 3.3.1. Phase Analysis

**Figure 6:**
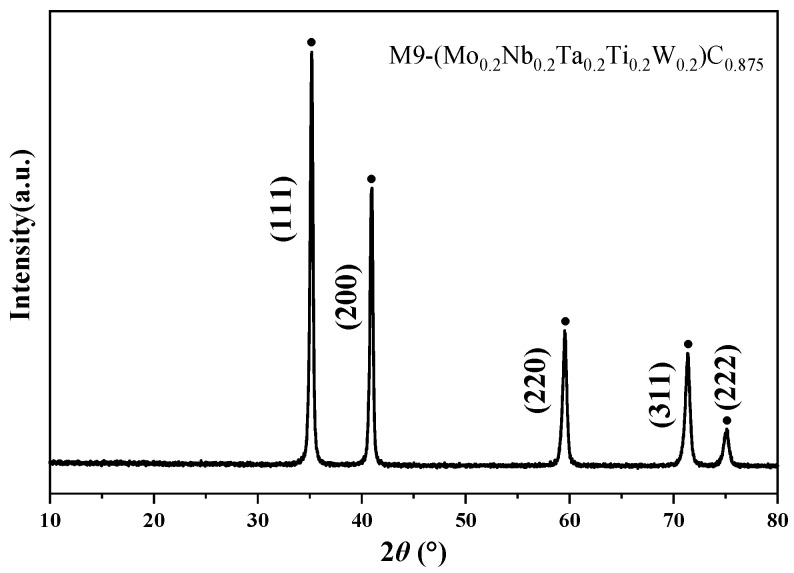
XRD pattern of M9 ceramic.

The XRD pattern of a ceramic block prepared from the M9 HEC powder via SPS is displayed in Figure 6. This diffraction pattern only exhibits the characteristic diffraction peaks of an FCC rock-salt structure phase, and no obvious diffraction peaks of impurity phases or secondary phases are detected. Therefore, the crystal structure of the M9 powder remains stable after the SPS process and does not undergo any phase transformation. This result confirms that the single-phase rock-salt structure of the original powder is maintained during the preparation of bulk HEC ceramics via SPS [37].

#### 3.3.2. Microstructural Analysis

The relative density and average grain size of the M9 HEC ceramic block prepared via SPS are summarized in Table 4. Based on the XRD analysis, the calculated lattice constant of the M9 block is 0.4465 nm, which is significantly larger than that of the original powder (0.4383 nm). The significantly enhanced interdiffusion of different metal atoms (Nb, Ti, Ta, Mo, and W) during the high-temperature sintering process likely explains this lattice expansion phenomenon. SPS promotes the formation of a solid solution and brings the reaction system closer to thermodynamic equilibrium, resulting in more complete solid solution formation [38]. In other words, the increase in the lattice constant is a result of the synergistic effect of increased solid solubility and lattice distortion [39]. The theoretical density of the M9 ceramic block is 10.37 g/cm^3^, and the measured density of this ceramic is as high as 99.1%. Therefore, near-complete dense ceramic sintering was achieved during the SPS process.

Microstructural analysis (SEM) revealed that the average grain size of the M9 ceramic block is 4.3 μm. Compared to the initial nanoscale powder, the sintering process induces significant grain growth. This inevitably occurs during high-temperature sintering due to grain boundary migration and grain coarsening. Compared to similar materials prepared under similar sintering temperatures and pressures as reported in the literature [20], the “preparing nanoscale powder first and then sintering with SPS” approach utilized in this study achieves a higher relative density. This result highlights the advantage of using nanoscale powders prepared via the carbothermic reduction of oxides as a precursor to promote the densification of HECs.

**Figure 7:**
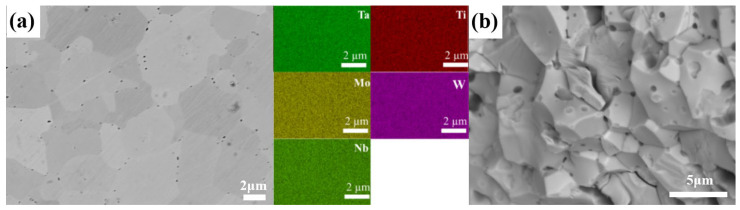
(**a**) SEM image and EDS mapping of M9 ceramic; (**b**) SEM image of fracture surface of M9 ceramic.

An SEM image and corresponding EDS mapping analysis of the M9 HEC ceramic block are shown in Figure 7. Figure 7a reveals the high overall density of the sintered M9 ceramic block. However, a small amount of residual porosity is observed in localized areas, which are primarily distributed at the grain boundaries. This is consistent with the pinning or trapping mechanism of pores during grain boundary migration in the later stages of sintering. The EDS elemental mapping clearly demonstrates that the five metal elements (Nb, Ti, Ta, Mo, and W) are uniformly distributed at the micrometer scale, with no evident elemental segregation or agglomeration [40]. The slight contrast variations in the BSE image are attributed to crystallographic orientation effects and not compositional inhomogeneity, a conclusion supported by the uniform EDS maps. This confirms the good compositional homogeneity of this bulk ceramic material on a larger scale, corroborating the nanoscale TEM-EDS analysis (Figure 5). Figure 7b displays an SEM image showing the typical fracture morphology of the M9 ceramic block. Clear and complete grain contours are visible on the fracture surface, indicating that the fracture mode of the material is primarily intergranular fracture [41]. Materials that exhibit fracture behavior typically have relatively weaker grain boundary strengths compared to the interior of the grains. In conjunction with the uniform elemental distributions shown in Figure 7a, it is speculated that the intergranular fracture observed in this study may be related to the residual pores or inherent grain boundary characteristics rather than grain boundary segregation being dominant.

#### 3.3.3. Mechanical Performance

The mechanical properties of the M9 ceramic sample are presented in Table 5. M9 high-entropy carbide ceramics exhibit excellent mechanical properties. Its Vickers hardness and nano-hardness reach 17.3 GPa and 25.9 GPa, respectively, with a fracture toughness as high as 4.43 MPa·m^1/2^. These properties are significantly superior to those of most single-component and binary carbide or boride ceramics. The outstanding mechanical properties of the (Mo_0.2_Nb_0.2_Ta_0.2_Ti_0.2_W_0.2_)C_0.9_ ceramic can be attributed to its specific elemental composition. The strategic inclusion of W, instead of the more commonly studied Zr or V, plays a pivotal role. The atomic radius of W (≈1.39 Å) is significantly smaller than that of Zr (≈1.60 Å), which increases the atomic size mismatch within the cationic sublattice. This heightened mismatch exacerbates lattice strain and distortion, leading to more potent solid-solution strengthening. This effect is a primary contributor to the high hardness observed. The higher fracture toughness may be due to the lattice distortion effect and the hindrance of crack propagation by the intergranular fracture mode. At the same time, the fine-grained microstructure obtained by carbothermic reduction combined with SPS technology also contributes to the strengthening and toughening effect. The (Mo_0.2_Nb_0.2_Ta_0.2_Ti_0.2_W_0.2_)C_0.9_ ceramic exhibits favorable mechanical properties (hardness: 17.3 GPa, fracture toughness: 4.43 MPa·m^1/2^). Compared to the (MoNbTaTiW)C_4.5_ ceramic [20] (fracture toughness: 5.2 MPa·m^1/2^; relative density: 97.2%; grain size: 6.5 μm), the present material achieves higher densification (99.1%) and finer grains (4.3 μm) due to optimized powder synthesis. These comparisons underscore the significance of precursor powder quality and stoichiometric control in determining HEC properties. To better contextualize the performance of the synthesized (Mo_0.2_Nb_0.2_Ta_0.2_Ti_0.2_W_0.2_)C_0.9_ ceramic, its mechanical properties are compared with those of several well-established high-performance ceramics in Table 5. Although the Vickers hardness of our HEC ceramic (17.3 GPa) is lower than that of pure WC (24–25 GPa) [42] and TiC (22–28 GPa) [43], it exceeds that of Al_2_O_3_ (15–20 GPa) [44] and YSZ (12–13 GPa) [45]. More notably, the fracture toughness of our material (4.4 MPa·m^1/2^) is higher than that of TiC (3.5–4.5 MPa·m^1/2^) [43] and comparable to Al_2_O_3_ (3–5 MPa·m^1/2^) [44], though it remains lower than that of YSZ (6–10 MPa·m^1/2^) [45]. Furthermore, the Young’s modulus of our ceramic (524 GPa) is exceptionally high, significantly surpassing that of Al_2_O_3_ (380–400 GPa) [44] and YSZ (210–220 GPa) [45] and approaching that of WC (~600 GPa) [42]. This combination of high hardness, moderate fracture toughness, and outstanding stiffness demonstrates the potential of nonstoichiometric high-entropy carbides as promising candidates for structural applications under extreme conditions.

The thermal conductivity values of the M9 HEC ceramic block at different temperatures are presented in Table 6. At room temperature, the M9 ceramic has a relatively low thermal conductivity of only 8.3 W·m^−1^·K^−1^. This low thermal conductivity can be attributed to the significant high-entropy lattice distortion effect [46]. The structure of this ceramic contains multiple cations (Mo, Nb, Ta, Ti, and W) with large differences in atomic radius, and the random occupation of lattice sites by these different cations leads to strong local lattice strain and distortion. This atomic-scale structural disorder significantly enhances phonon scattering and increases the thermal resistance of the lattice, leading to a significant reduction in thermal conductivity [17]. It is worth noting that thermal conductivity increases with increasing temperature, because ceramics are insulator materials with thermal conduction properties that mainly rely on the propagation of lattice vibrational waves (phonons). As the temperature increases, an increasing number of lattice vibrational modes are excited, resulting in an increase in the apparent thermal conductivity [47].

## 4. Conclusions

In this study, nonstoichiometric high-entropy carbide (Mo_0.2_Nb_0.2_Ta_0.2_Ti_0.2_W_0.2_)C_x_ nanometer powders were successfully synthesized via the carbothermic reduction of precursor oxides, and dense ceramic blocks were prepared using spark plasma sintering. The main conclusions are as follows:

1. Using carbothermic reduction, HEC nanoparticles with a single-phase rock-salt structure are formed at temperatures of 1500 °C and above, and the average particle size of the prepared powders is as low as 270 nm. The five metal elements (Nb, Ti, Ta, Mo, and W) of the HEC powders are uniformly distributed on the nanoscale and exhibit good crystallization. The lattice constant increases with decreasing carbon content and sintering temperature.
2. The HEC ceramic prepared by ball milling the optimal HEC powder followed by SPS at 1950 °C has a relative density of 99.1% and an average grain size of 4.3 μm. This ceramic has a Vickers hardness of 17 GPa, a nano-hardness of 26 GPa, a Young’s modulus of 524 GPa, and a fracture toughness of 4.4 MPa·m^1/2^. The room-temperature thermal conductivity of this ceramic is only 8.3 W/m·K.

The exceptional combination of properties demonstrated by the (Mo_0.2_Nb_0.2_Ta_0.2_Ti_0.2_W_0.2_)C_0.9_ ceramic suggests significant potential for applications in extreme environments. Specifically, this material is a promising candidate for use as next-generation cutting tools, wear-resistant coatings, and thermal barrier coatings in aerospace and energy systems, where simultaneous mechanical integrity and thermal insulation are critical. Its stability and performance at high temperatures warrant further investigation for these purposes.

## Figures and Tables

**Table 1:** Composition and preparation process of (Mo_0.2_Nb_0.2_Ta_0.2_Ti_0.2_W_0.2_)C_x_ high-entropy powders.

No.	Nominal Chemical Formula	Molar Ratio of MoO_3_, Nb_2_O_5_, Ta_2_O_5_, TiO_2_, and WO_3_ to C	Reaction Temperature/°C
M1	(Mo_0.2_Nb_0.2_Ta_0.2_Ti_0.2_W_0.2_)C	1:0.5:0.5:1:1:18	1550
M2	(Mo_0.2_Nb_0.2_Ta_0.2_Ti_0.2_W_0.2_)C_0.972_	1:0.5:0.5:1:1:17.5	1600
M3	(Mo_0.2_Nb_0.2_Ta_0.2_Ti_0.2_W_0.2_)C_0.972_	1:0.5:0.5:1:1:17.5	1550
M4	(Mo_0.2_Nb_0.2_Ta_0.2_Ti_0.2_W_0.2_)C_0.972_	1:0.5:0.5:1:1:17.5	1500
M5	(Mo_0.2_Nb_0.2_Ta_0.2_Ti_0.2_W_0.2_)C_0.924_	1:0.5:0.5:1:1:16.63	1550
M6	(Mo_0.2_Nb_0.2_Ta_0.2_Ti_0.2_W_0.2_)C_0.924_	1:0.5:0.5:1:1:16.63	1500
M7	(Mo_0.2_Nb_0.2_Ta_0.2_Ti_0.2_W_0.2_)C_0.924_	1:0.5:0.5:1:1:16.63	1400
M8	(Mo_0.2_Nb_0.2_Ta_0.2_Ti_0.2_W_0.2_)C_0.875_	1:0.5:0.5:1:1:15.75	1550
M9	(Mo_0.2_Nb_0.2_Ta_0.2_Ti_0.2_W_0.2_)C_0.875_	1:0.5:0.5:1:1:15.75	1500
M10	(Mo_0.2_Nb_0.2_Ta_0.2_Ti_0.2_W_0.2_)C_0.875_	1:0.5:0.5:1:1:15.75	1400

**Table 2:** Critical temperature of carbothermic reduction of five oxides with carbon (*p* = 50 Pa).

Reaction System	(MoO_3_ + C)	(WO_3_ + C)	(Nb_2_O_5_ + C)	(Ta_2_O_5_ + C)	(TiO_2_ + C)
Critical temperature/K	540.15	658.15	905.15	1005.15	1145.15

**Table 3:** Lattice parameters of (Mo_0.2_Nb_0.2_Ta_0.2_Ti_0.2_W_0.2_)C_x_ powders.

No.	M1	M2	M3	M4	M5	M6	M7	M8	M9	M10
Lattice parameter/nm	0.4378(1)	0.4377(1)	0.4379(1)	0.4381(1)	0.4380(2)	0.4384(1)	0.4395(2)	0.4381(1)	0.4383(1)	0.4395(2)

(1) denotes ±0.0001 nm; (2) denotes ±0.0002 nm.

**Table 4:** Density and grain size of M9 ceramic.

Lattice Parameter /nm	Theoretical Density /g/cm^3^	Measured Density /g/cm^3^	Relative Density /%	Grain Size /μm
0.4465(1)	10.37	10.28 ± 0.01	99.1 ± 0.2	4.3 ± 1.3

0.4465(1) represents 0.4465 ± 0.0001 nm.

**Table 5:** Comparison of the hardness, Young’s modulus, and fracture toughness of various ceramic materials.

Material	Vicker’s Hardness /GPa	Young’s Modulus /GPa	Fracture Toughness /MPa·m^1/2^	References
(Mo_0.2_Nb_0.2_Ta_0.2_Ti_0.2_W_0.2_)C_0.9_	17.3 ± 1.3	524 ± 29	4.4 ± 0.4 ^1^	This work.
WC (pure, hot-pressed)	24–25	~600	5.7–6.7	[42]
TiC	22–28	~450–480	3.5–4.5	[43]
Al_2_O_3_	15–20	380–400	3–5	[44]
YSZ (Yttria-Stabilized Zirconia)	12–13	210–220	6–10	[45]

^1^ In the Supplementary Materials, we have included SEM images that show the Vickers marks and related radial cracks on the polished surface of the M9 ceramic sample.

**Table 6:** Thermal conductivity of the M9 ceramic sample at different temperatures.

Temperature /°C	25	200	400	600	800	1000	1200
Thermal conductivity /W/m·K	8.49 ± 0.07	11.48 ± 0.01	13.88 ± 0.01	16.49 ± 0.02	18.57 ± 0.01	20.63 ± 0.22	22.94 ± 0.70

## Data Availability

The original contributions presented in this study are included in the article or Supplementary Material. Further inquiries can be directed to the corresponding author.

## Supplementary Materials

The following supporting information can be downloaded at https://www.mdpi.com/article/10.3390/ma18184293/s1: Figure S1: (a) The indentation crack propagation diagram of the M9 ceramic sample and (b) diagrammatic sketch of the measurement on the crack length for KIC.
