# Supplementary material for: Synthesis and Sintering of Nonstoichiometric (Mo0.2Nb0.2Ta0.2Ti0.2W0.2)Cx Nanoscale Powders for Highly-Dense Ceramics
Source: Materials (Basel). 2025 Sep 12;18(18):4293. doi: 10.3390/ma18184293 (PMC12471743; doi:10.3390/ma18184293)
Supplement: Supplementary file 1 [file materials-18-04293-s001.zip › materials-3834704-supplementary.pdf]

materials

MDPI

Supplementary materials

Synthesis and Sintering of Non-Stoichiometric $(Mo_{0.2}Nb_{0.2}Ta_{0.2}Ti_{0.2}W_{0.2})C_x$ Nanoscale Powders for Highly-Densed Ceramics

Wanxiu Hai [1,2,3,*], Hai Zhang [1], Liulin Li [1], Tong He [1], Shubo Zhang [1], Meiling Liu [1,2,3], Yuhong Chen [1,2], Youjun Lu [1,3] and Hailong Wang [4]

[1] College of Materials Science & Engineering, North Minzu University, Yinchuan 750021, China; 13575334559@163.com (H.Z.); 17309475821@163.com (L.L.); 15719510669@163.com (T.H.); matinal757791@163.com (S.Z.)
[2] Key Laboratory of Powders & Advanced Ceramics, North Minzu University, Yinchuan 750021, China
[3] Laboratory of Helan Moutain, Yinchuan 750021, China
[4] College of Materials Science & Engineering, Zhengzhou University, Zhengzhou 450001, China; 119whl@zzu.edu.cn
* Correspondence: wxhai@nun.edu.cn

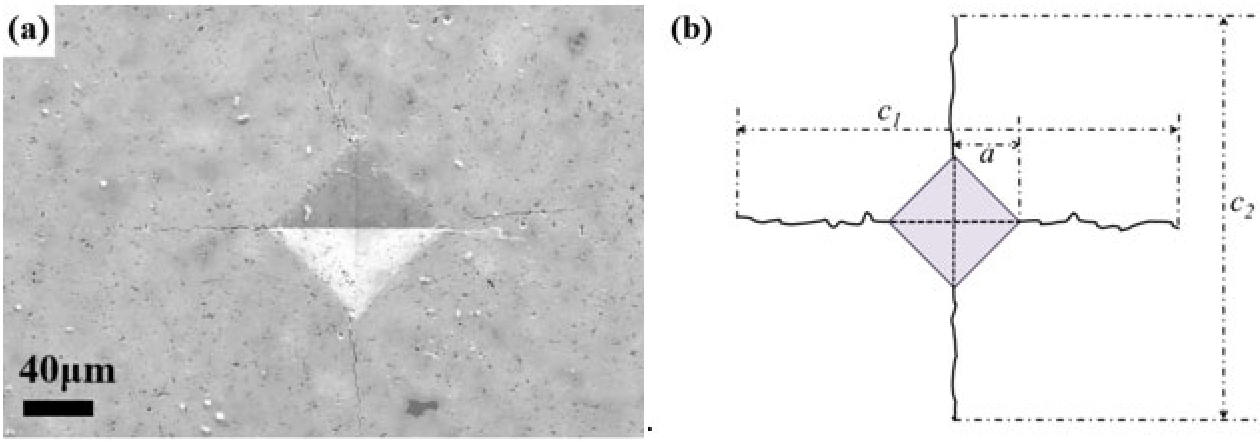

Figure S1. (**a**) The indentation crack propagation diagram of the M9 ceramic sample and (**b**) diagrammatic sketch of the measurement on the crack length for KIC.

The fracture toughness is measured using the Vickers hardness indentation method. After unloading, four cracks propagating from the four corners of the indentation were observed under microscopy, as shown in Figure S1(a). The lengths of the indentation and cracks were measured. With reference to the schematic diagram of crack length measurement illustrated in Figure S1(b), the fracture toughness KIC of the sample was calculated using Equation (7) provided in the main text. In the above equation, E represents the measured Young's modulus of the sample, Hv is the Vickers hardness value of the sample under the test load, P is the indentation load, a is half of the indentation length, and c is the average value of the two radial cracks produced by the same indentation, that is, $c = (c_1 + c_2) / 2$.

These images clearly display the indent and the crack lengths that were measured to calculate the KIC value of 4.43 ± 0.4 MPa·m$^{1/2}$.
